# Non-*albicans Candida* Infection: An Emerging Threat

**DOI:** 10.1155/2014/615958

**Published:** 2014-10-22

**Authors:** Sachin C. Deorukhkar, Santosh Saini, Stephen Mathew

**Affiliations:** Department of Microbiology, Rural Medical College, Pravara Institute of Medical Sciences (Deemed University), Loni, Maharashtra 413736, India

## Abstract

The very nature of infectious diseases has undergone profound changes in the past few decades. Fungi once considered as nonpathogenic or less virulent are now recognized as a primary cause of morbidity and mortality in immunocompromised and severely ill patients. *Candida* spp. are among the most common fungal pathogens. *Candida albicans* was the predominant cause of candidiasis. However, a shift toward non-*albicans Candida* species has been recently observed. These non-*albicans Candida* species demonstrate reduced susceptibility to commonly used antifungal drugs. In the present study, we investigated the prevalence of non-*albicans Candida* spp. among *Candida* isolates from various clinical specimens and analysed their virulence factors and antifungal susceptibility profile. A total of 523 *Candida* spp. were isolated from various clinical specimens. Non-*albicans Candida* species were the predominant pathogens isolated. Non-*albicans Candida* species also demonstrated the production of virulence factors once attributed to *Candida albicans*. Non-*albicans Candida* demonstrated high resistance to azole group of antifungal agents. Therefore, it can be concluded that non-*albicans Candida* species have emerged as an important cause of infections. Their isolation from clinical specimen can no longer be ignored as a nonpathogenic isolate nor can it be dismissed as a contaminant.

## 1. Introduction

Over the last few years, the incidence of mycotic infections has progressively increased. Fungi once considered as nonpathogenic or less virulent are now recognized as a primary cause of morbidity and mortality in immunocompromised and severely ill patients [1].

*Candida* spp. are among the most common fungal pathogens. They are capable of initiating infections in both immunocompetent individuals and immunocompromised hosts, but the incidence of infections is more in immunocompromised individuals; candidiasis, hence, is rightly called the “disease of diseased” [2].

*Candida* spp., though commensal organisms that normally colonize mucosal surfaces in an asymptomatic manner, can become one of the most significant causes of disabling and lethal infection [3].* Candida* spp. are responsible for various clinical manifestations ranging from mucocutaneous overgrowth to life threatening disseminated infections like candidemia [4].

Although* Candida albicans* is the most prevalent species involved in both mucocutaneous and disseminated infections, the incidence of candidiasis due to non-*albicans Candida* (NAC) spp. is increasing [4]. Several factors like severe immunosuppression or illness, prematurity, use of broad spectrum antibiotics, and empirical use of antimycotic drugs are reported to be associated with this change. The clinical manifestations of infections caused by different members of NAC spp. are usually indistinguishable, but several NAC spp. are inherently resistant or acquire resistance, or both, to commonly used antifungal drugs [5].

The transition of* Candida* spp. from commensal to potent pathogen is facilitated by a number of virulence factors such as adherence to host tissues and medical devices, biofilm formation, and secretion of extracellular hydrolytic enzymes [4]. Although there has been extensive research to identify these pathogenic attributes in* C. albicans*, relatively less is known about NAC spp. [6].

The present study was conducted at a rural tertiary care teaching hospital of India with an aim of investigating prevalence of NAC spp. among* Candida* isolates from various clinical specimens and study their virulence factors and antifungal susceptibility profile.

## 2. Materials and Methods

The present study is part of a Ph.D. thesis conducted in the Department of Microbiology, Rural Medical College and Hospital of Pravara Institute of Medical Sciences, Loni, Maharashtra, India. The protocol of the study was approved by the Institutional Ethics Committee.* Candida *spp. isolated from various clinical specimens between the period of January 2010 and December 2013 were included in the study.

The repeated isolation of* Candida* spp. from clinical specimens collected from oropharyngeal, vaginal, urinary, and bronchial candidiasis was considered significant, while a single isolation was considered significant from sterile body fluids like blood, peritoneal fluid, pleural fluid, and cerebrospinal fluid (CSF).

Patient's information such as date of admission, ward, underlying medical conditions, associated risk factors such as presence of urinary catheter, respiratory ventilation, central line insertion, duration of antibiotic therapy, antifungal prophylaxis, exposure to invasive medical procedures, and use of corticosteroids was obtained from clinical records and analysed.

Colonies appearing pasty, opaque, slightly domed or flat, smooth and pale coloured (white, off-white, or beige) with a sweet smell reminiscent of ripe apples were suspected to be colonies of* Candida* [7]. The suspected colonies of* Candida* isolates were identified by wet film, Gram stain, and India ink preparation.

### 2.1. Species Identification

The mycological workup for speciation of* Candida* isolates started with the germ tube test. Isolates producing germ tubes within 2 hours of incubation were further subjected to temperature studies, chlamydospores formation, and biochemical tests for differentiation of* C. albicans* from* C. dubliniensis*. Germ tube negative* Candida *isolates were classified on the basis of sugar assimilation and colony colour on Hichrom* Candida* agar. HiCandida Identification Kit (HiMedia Laboratories Pvt. Ltd., Mumbai, India) supplemented the identification of isolates.

### 2.2. Virulence Factors

*Candida* spp. isolated from various clinical specimens were screened for the production of virulence factors such as adherence to buccal epithelial cell (ABEC), biofilm formation, haemolytic activity, and production of extracellular hydrolytic enzymes (coagulase, phospholipase, and proteinase). For each virulence factor, the isolate was tested in triplicate in each assay and three assays were carried out for each isolate on separate occasions. The mean value obtained was considered for analysis.

#### 2.2.1. Adherence to Buccal Cell (ABEC)

Adherence assay was performed as described by Kimura and Pearsall [8] with minor modifications. BECs were collected by gently rubbing the cheek mucosa of eight (four males, four females) healthy laboratory technicians (no signs or symptoms of OPC or other oral lesions and not receiving any antibiotics at the time of study) after obtaining prior consent. As fresh BECs were used, they were collected in the morning on the day of assay. BECs were washed thrice by phosphate buffered saline (PBS) and harvested by centrifugation.

Equal volumes (1 mL) of BEC (1 × 10^5^ cells/mL) and yeast suspension (1 × 10^7^ cells/mL) were mixed and incubated at 37°C for 2 h in a shaking water bath at 40 rpm. The mixture was filtered through a 20 *μ*m filter to remove nonadherent yeast cells. The BECs on the filter were washed with 5 mL of PBS and finally suspended in 5 mL of PBS. A drop of this suspension was placed on glass slide. The smear was fixed by methanol, air-dried, and stained with 2% crystal violet for 1 minute. Adherence was determined microscopically by counting the mean number of yeast cells adhering to every 100 BECs.* C. albicans* ATCC 90028 was used as the control strain.

#### 2.2.2. Biofilm Formation

Biofilm formation was determined using the tube method described by Yigit et al. [9] with a few modifications.* C. albicans* ATCC 90028 and* C. albicans* ATCC 10231 were used as control strains.

Colonies of* Candida* isolates to be tested for biofilm formation were inoculated in saline and incubated for 24 h at 35°C. 1.5 mL of this saline suspension was transferred to screw capped conical polystyrene tubes containing 5 mL of Sabouraud dextrose broth supplemented with glucose (final concentration, 8%). The tubes were incubated at 37°C for 24 h without agitation.

After incubation, the broth from the tube was gently aspirated using a Pasteur pipette. The tube was washed thrice with PBS (pH 7.2) and stained with 1% safranin. The stain was decanted after 15 min and the tube was rinsed with PBS to remove excess stain.

Presence of visible adherent film on the wall and the bottom of the tube indicated biofilm formation by the isolate. Ring formation at the liquid interface was not considered as an indication of biofilm production.* C. albicans* ATCC 90028 and* C. albicans* ATCC 10231 were used as control strains.

#### 2.2.3. Haemolytic Activity

Haemolytic activity of* Candida* spp. was screened by the method described by Luo et al. [10]. Approximately 10 *μ*L of standard inoculum (10^8^ *Candida* cells/mL) was inoculated on sheep blood Sabouraud dextrose agar plate. Plates were incubated at 37°C in 5% CO_2_ for 48 h. The presence of a distinct translucent halo around the inoculum site, viewed with transmitted light, indicated haemolysin production. Haemolytic activity (Hz) was determined by calculating the ratio of the diameter of the colony to that of the translucent zone of haemolysis.* C. albicans* ATCC 90028 and* C. parapsilosis* ATCC 22019 were used as positive and negative controls, respectively.* Streptococcus pyogenes* (Lancefield group A) and* Streptococcus sanguis* were used as positive controls for beta and alpha haemolysis, respectively.

#### 2.2.4. Production of Extracellular Hydrolytic Enzymes

The* Candida* isolates were screened for the production of exoenzymes like coagulase, phospholipase, and proteinase.

*(i) Coagulase Production*. Coagulase production was assessed by the method described by Rodrigues et al. [11]. Approximately 0.1 mL of an overnight culture of* Candida* spp. was aseptically inoculated into a test tube containing 0.5 mL of EDTA-rabbit plasma. The tube was incubated at 35°C and observed for clot formation after 4 h. The presence of clot that could not be resuspended by gentle shaking indicated positive coagulase test. If no clot formed, the tube was reincubated and reexamined at 24 h.* Staphylococcus aureus* ATCC 25923 and* S. epidermidis* ATCC 14990 were used, respectively, as positive and negative controls.

*(ii) Phospholipase Production*. Phospholipase production was assayed according to the method of Samaranayake et al. [12] by egg yolk agar plate method. Approximately 5 *μ*L of standard inoculum of test strain containing 10^8^ cells/mL was aseptically inoculated onto egg yolk agar. After inoculation, the plates were dried at room temperature and then incubated at 35°C for 3 days. The plates were examined for the presence of a zone of precipitate around the colony (phospholipase production).

Phospholipase activity (Pz) was expressed as the ratio of the colony diameter to the diameter of the colony plus the precipitation zone. A Pz value of 1 denoted no phospholipase activity, whereas Pz < 1 indicated phospholipase expression by the isolate.* C. albicans* ATCC 10231 was used as the positive control.

*(iii) Proteinase Production*. Proteinase production was performed according to the method of Aoki et al. [13] with a few modifications, using bovine serum albumin agar (BSA) plates. Approximately 10 *μ*L of standard inoculum containing 10^6^ cells/mL was aseptically inoculated onto 1% BSA agar plate. Inoculated plates were incubated at 37°C for 7 days. Further proteinase activity was inhibited by adding 20% trichloroacetic acid and the plate was stained with 1.25% amido black. The diameter of the colonies was measured prior to staining and the diameter of the clear zones was measured after staining.

Proteinase index (Prz) was measured in terms of the ratio of the diameter of the colony to the diameter of unstained zone. A Prz value of 1 indicated no proteinase activity; Prz < 1 denoted proteinase expression by* Candida* isolate. The lower the Prz value, the higher the activity.* C. albicans* ATCC 10231 was used as positive control.

### 2.3. Antifungal Susceptibility Testing

In vitro antifungal susceptibility testing of* Candida *isolates was performed using HiComb MIC test (HiMedia Laboratories Pvt. Ltd., Mumbai, India). Instructions provided by the manufacturer were adhered to throughout the test. The antifungals tested in this study were amphotericin B (range 0.002–32 *μ*g), fluconazole (range 0.016–256 *μ*g), itraconazole (range 0.002–32 *μ*g), and ketoconazole (range 0.002–32 *μ*g).

The inoculum was prepared by inoculating 3-4 colonies of the* Candida* isolate in saline. The turbidity of the suspension was matched to 0.5 McFarland standard. The yeast suspension was inoculated on Mueller-Hinton agar supplemented with 2% glucose and methylene blue (0.5 *μ*g/mL) by the lawn culture method using a tipped cotton swab. The inoculum was allowed to dry and the strip was placed on the surface of agar with the help of forceps. The plates were incubated at 35°C 24–48 h.* C. albicans* ATCC 90028 and* C. parapsilosis* ATCC 22019 were used for quality control.

The results of antifungal susceptibility test were interpreted as sensitive (S), susceptible dose dependent sensitive (SDD), and resistant (R). Interpretative criteria for azoles were those recommended by the Clinical and Laboratory Standard Institute (CLSI) [14, 15]. Due to the lack of defined breakpoints for amphotericin B, arbitrary values based on the studies of other researchers were used [16, 17].

## 3. Results

**Figure 1:**
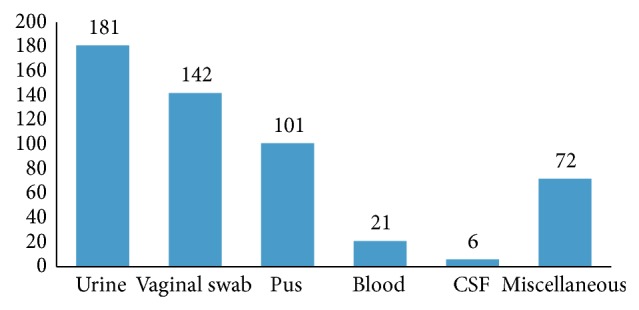
Clinical samplewise distribution of* Candida* isolates.

Between January 2010 and December 2013, 523* Candida* spp. were isolated from various clinical specimens. The distribution of* Candida* spp. in various clinical specimens is shown in Figure 1. The majority of* Candida* spp. were isolated from urine (34.6%) followed by vaginal swabs (27.1%) and oropharyngeal swabs (19.3%).

Out of 523* Candida* spp. isolated from various clinical specimens, 192 (36.7%) were* C. albicans* and 331 (63.3%) were NAC spp. Among the NAC spp.,* C. tropicalis* (35.1%) followed by* C. glabrata* (28.1%) and* C. krusei* (16.3%) was the major isolates. Out of 9* C. dubliniensis*, 7 were isolated from oropharyngeal swabs collected from HIV infected patients with oropharyngeal candidiasis (OPC), whereas 2 were isolated from vaginal swabs collected from HIV noninfected clinically suspected cases of vulvovaginal candidiasis (VVC).* C. glabrata* was the major isolate from cases of candidemia followed by* C. tropicalis* and* C. albicans* (Table 1).

Table 2 shows virulence factors produced by* Candida* isolates. Phospholipase production followed by haemolysin production and ABEC were the major virulence factors produced by* C. albicans*. Among NAC spp.,* C. tropicalis* (63.7%) followed by* C. glabrata* (60.2%) showed maximum adherence to BEC. Biofilm formation capacity was higher in* C. tropicalis* (74.1%) as compared to* C. albicans* (72.9%). Coagulase production was not seen in* C. kefyr* and* C. dubliniensis* isolates. Phospholipase activity was higher among* Candida* isolates capable of producing biofilms.* C. tropicalis* followed by* C. glabrata* showed maximum phospholipase activity, whereas proteinase production was high in* C. tropicalis* and* C. dubliniensis* isolates.

Antifungal susceptibility profile of* Candida* isolates is shown in Table 3. Amphotericin B resistance was more common in* C. albicans* as compared to NAC spp. Resistance to the azole group of antifungal agents was common in NAC spp.* C. dubliniensis* followed by* C. glabrata* demonstrated high resistance to fluconazole. Itraconazole resistance was more common in* C. glabrata* and* C. krusei*. Ketoconazole resistance was high in* C. dubliniensis* and* C. glabrata* isolates.

## 4. Discussion

The very nature of infectious diseases has undergone profound changes in the past few decades. Hitherto unknown microbes or microorganisms with no pathogenic role have emerged as important causes of morbidity and mortality worldwide. In recent years,* Candida* spp. have emerged as principal pathogens of a variety of human infections.

In the present study, the majority of* Candida *spp. (181) were isolated from urine samples. Of these, 71 (39.2%) were* C. albicans,* while 110 (60.8%) isolates belonged to NAC spp.* C. tropicalis* followed by* C. glabrata* was the most prevalent isolate from NAC group. Our observation is similar to that of Álvarez-Lerma et al. [18] and Kauffmann [19], where >50% of urinary* Candida* isolates belonged to NAC spp. NAC spp. are not only well adapted to the urinary tract but also more difficult to eradicate than* C. albicans*. Presence of indwelling urinary catheters, advanced age, diabetes mellitus, and pregnancy were major risk factors associated with candiduria. Incidence of candiduria was high among patients admitted to the ICU and among those who had a previous history of treatment with antibiotics. The abuse of antibiotics as a “pill for all ills,” self-medication, and starting broad spectrum antibiotics as the first line treatment have led to increased colonization by* Candida* spp. which suppressed the commensal bacterial flora.

OPC and VVC are the most common features of mucosal candidiasis. VVC though an extremely common infection in women of childbearing age has been now excluded from the list of sexually transmitted infections, contributing to a dearth of recent information regarding its incidence and epidemiology [20]. The diagnosis of VVC is usually made on the basis of clinical examination with minimal or no laboratory support. In our study, 66.3% of* Candida* spp. isolated from VVC cases belonged to NAC spp.* C. glabrata* and* C. tropicalis* were predominant pathogens. This observation is in accordance with studies by Mohanty et al. [21] and Jindal et al. [22]. Low dosage azole maintenance regimen, uncontrolled diabetes mellitus, and douching are the most common risk factors identified for VVC due to* C. glabrata*. In our study,* C. dubliniensis* was recovered from 2 HIV negative VVC patients. This observation confirms the possibility of* C. dubliniensis* infection in a population other than the HIV infected.

OPC is the most common opportunistic mycoses in immunocompromised individuals. In our study, NAC spp. were the predominant pathogens isolated from OPC cases. Widespread use of immunosuppressive therapy and broad spectrum antimycotic prophylaxis has increased the incidence of OPC due to NAC spp.

*C. glabrata *has been increasingly reported in disseminated infections in recent years [2]. In our study,* C. glabrata* was the predominant species of* Candida* isolated from cases of candidemia. The risk factors leading to* C. glabrata* blood stream infection are similar to those by other species, but, compared to other* Candida *spp., the mortality rate of* C. glabrata* infection is high [2].

In* Candida* spp., the transition from commensal to potential pathogen is determined by various host predisposing factors and virulence attributes of infecting species [4]. Identifying these virulence factors in infecting pathogens and understanding their effects on the human host are a major challenge for clinical microbiologists. Adhesion of* Candida* spp. to the host epithelial cells is a critical first step in the pathogenesis of infection. Binding of the* Candida* to host cells, host cell proteins, or microbial competitors prevents or at least reduces the extent of clearance by the host's defense mechanisms [23]. ABEC was highest in* C. albicans*. A similar observation was reported by Mane et al. [24]. Among NAC spp.,* C. tropicalis* followed by* C. glabrata* and* C. dubliniensis* demonstrated high adherence to buccal epithelial cells.

The increasing incidence of hospitalization, advances in medical science, increasing use of antimicrobial agents accompanied by better adaptation of microorganisms to the hospital environment, all these factors, have combined to increase health care associated infections (HCAIs). Due to their versatility of adapting to a variety of different habitats including various medical devices,* Candida *spp. have emerged as one of the leading causes of HCAIs.* Candida* spp. possess the ability to form biofilm on most, if not all, medical devices [25]. Biofilms are surface-associated communities of microorganisms embedded within an extracellular matrix [26]. In this study, we noted greater biofilm forming ability in* C. tropicalis* as compared to* C. albicans*. Biofilm formation is implicated as an important virulence attribute of* Candida* spp. as it increases the ability to withstand host defenses and also confers significant resistance to antifungal therapy [4]. It also aids in establishing a reservoir for continuing infections. Biofilm forming strains are associated with higher morbidity and mortality rates. The formation of mature biofilm and subsequent production of extracellular matrix are strongly dependent on species, strain, and environmental condition [26].

In* Candida, *extracellular hydrolases play an important role in adherence, tissue penetration, invasion, and the destruction of host tissue [4]. Therefore, production of hydrolytic enzymes is one of the important attributes contributing to pathogenesis of* Candida*. Although* Candida* is capable of producing exoenzymes, the quantity and potency of these enzymes are different. Production of extracellular hydrolases varies among the species and also depends on the source or site of infection [27].

Enzyme coagulase binds plasma fibrinogen and activates a cascade of reactions that induce clotting of plasma [9]. In our study, coagulase production was high in* C. albicans* as compared to NAC spp. Among NAC spp.,* C. glabrata* showed high coagulase expression. Our observation is similar to that of Yigit et al. [9].

Of various hydrolytic enzymes produced by* Candida *spp., phospholipases and proteinases are the most important [4]. Phospholipases damage the host cell membrane and hence facilitate invasion of tissue [4]. These enzymes hydrolyze phospholipids into fatty acids and also expose receptors on host cell membrane to facilitate adherence [26]. In the present study, phospholipase production was high in* C. albicans* isolates. Phospholipase production was high in isolates from systemic candidiasis. Among NAC spp., phospholipase activity was highest in* C. tropicalis,* followed by* C. glabrata*. Expression of phospholipase enzyme was low in* C. dubliniensis*. This could be one of the possible reasons for minimal or no ability of* C. dubliniensis* to cause invasive infections. Phospholipase activity was high in biofilm forming* Candida* isolates. Screening of phospholipase activity in biofilm forming* Candida* spp. can be used as an important parameter to differentiate invasive strains from noninvasive colonisers [28].

Proteinase facilitates* Candida* invasion and colonization of host tissue by disruption of host membrane and by degrading important structural and immunological defense proteins [26]. In our study, although* C. albicans *demonstrated increased capability of proteinase production, significant proteinase activity was also noted in NAC spp. like* C. tropicalis*,* C. dubliniensis,* and* C. glabrata*.* C. dubliniensis* isolated from HIV infected patients with OPC demonstrated high proteinase activity.

Haemolysins are important for utilization of iron contained in haemoglobin. It can activate complement and opsonize surface of red blood cells [27]. They lead to destruction of host erythrocytes and facilitate hyphal invasion in systemic candidiasis [4, 9]. Therefore, haemolysin is considered as important virulence contributing to pathogenicity of* Candida* as it enables the pathogen to survive and persist in the host. In our study, haemolysin production was noted in both* C. albicans* and NAC spp.

Antifungal resistance once rarely documented has increased in recent years. The problem is compounded by aggressive immunosuppression (acquired or induced), an ageing population, and the emergence of virulent and intrinsically resistant organisms. In this study, amphotericin B resistance was less as compared to the azole group of antifungal agents. Azole resistance was more in NAC spp. as compared to* C. albicans*. Antifungal resistance was more common in* Candida* spp. isolated from systemic candidiasis and isolates producing virulence factors like biofilm. Though resistance to amphotericin B was less, this drug is often poorly tolerated and associated with acute infusion-related reactions and nephrotoxicity [29]. Resistance to azole group of antifungal agents can be due to quantitative or qualitative modifications of target enzymes, low access of the drug to the target, or a combination of these mechanisms [26]. Resistance to the azole group of antifungal agents is of concern because azoles like fluconazole are among the most commonly used antifungal agents for the treatment of candidiasis [29]. These drugs are safe and effective for the treatment of all clinical types of candidiasis. The broad use of triazoles, especially fluconazole, has given rise to concerns regarding the emergence of resistance [1]. Therefore, the task of identifying, isolating, and evaluating therapies of* Candida* species has now become part and parcel of clinical microbiology services.

## 5. Conclusion

Despite the advances in the field of medicine, infectious diseases continue to challenge mankind. During the last few decades, the spectrum of infections has undergone a drastic change; organisms with minimal or no pathogenic role have emerged as potent pathogens and organisms once susceptible have become multidrug resistant. In our study, NAC spp. were the predominant pathogens associated with various clinical types of candidiasis. Therefore, it can be concluded that NAC spp. have emerged as an important cause of infections. Its isolation from clinical specimens can no longer be ignored as nonpathogenic isolate nor can it be dismissed as a contaminant.

## Figures and Tables

**Table 1:** *Candida* spp. isolated from various clinical specimens.

*Candida* spp.	Urine	Vaginal swab	Oropharyngeal swab	Blood	CSF	Miscellaneous	Total
*C. albicans *	71	52	39	06	02	22	192
*C. tropicalis *	51	31	14	07	—	13	116
*C. glabrata *	24	41	11	08	04	05	93
*C. krusei *	14	11	08	—	—	21	54
*C. kefyr *	15	03	07	—	—	05	30
*C. parapsilosis *	04	—	09	—	—	05	18
*C. guilliermondii *	02	02	06	—	—	01	11
*C. dubliniensis *	—	02	07	—	—	—	09

Total	181	142	101	21	06	72	523

**Table 2:** Production of various virulence factors by *Candida* spp.

*Candida * spp.	ABEC (%)	Biofilm formation (%)	Coagulase production (%)	Haemolysin production (%)	Phospholipase production (%)	Proteinase production (%)
*C. albicans *	163 (83.1)	143 (72.9)	112 (57.1)	168 (85.7)	172 (87.7)	156 (79.5)
*C. tropicalis *	74 (63.7)	86 (74.1)	65 (56.1)	73 (62.9)	95 (81.8)	89 (76.7)
*C. glabrata *	56 (60.2)	59 (63.4)	45 (48.3)	62 (66.6)	61 (65.5)	56 (60.2)
*C. krusei *	16 (29.6)	12 (22.2)	8 (14.8)	13 (24.1)	12 (22.2)	12 (22.2)
*C. kefyr *	8 (26.6)	7 (23.3)	—	6 (20)	9 (30)	7 (23.3)
*C. parapsilosis *	4 (22.2)	5 (27.7)	3 (16.6)	4 (22.2)	6 (33.3)	5 (27.7)
*C. guilliermondii *	3 (27.2)	3 (27.2)	2 (18.1)	2 (18.1)	3 (27.2)	3 (27.2)
*C. dubliniensis *	5 (55.5)	—	—	3 (33.3)	1 (11.1)	6 (66.6)

**Table 3:** Antifungal susceptibility profile of *Candida* isolates.

*Candida* spp. (number of isolates)	Antifungal agent	*S* (%)	SDD (%)	*R* (%)
*C. albicans* (192)	Amphotericin B	164 (85.4)	8 (4.1)	20 (10.4)
	Fluconazole	121 (63.1)	6 (3.1)	65 (33.8)
	Itraconazole	116 (60.4)	2 (1.1)	74 (38.5)
	Ketoconazole	112 (45.3)	—	80 (41.7)

*C. tropicalis* (116)	Amphotericin B	98 (84.4)	9 (7.7)	9 (7.7)
	Fluconazole	69 (59.5)	3 (2.6)	44 (37.9)
	Itraconazole	65 (56.1)	2 (1.7)	49 (42.2)
	Ketoconazole	63 (54.3)	4 (3.5)	49 (42.2)

*C. glabrata* (93)	Amphotericin B	85 (91.3)	—	8 (8.7)
	Fluconazole	49 (52.7)	6 (6.5)	38 (40.8)
	Itraconazole	50 (53.8)	—	43 (46.2)
	Ketoconazole	51 (54.8)	2 (2.1)	60 (64.5)

*C. krusei* (54)	Amphotericin B	51 (94.4)	—	3 (5.6)
	Fluconazole	34 (62.9)	1 (1.9)	19 (35.2)
	Itraconazole	31 (57.4)	—	23 (42.6)
	Ketoconazole	32 (59.2)	2 (3.7)	20 (37.1)

*C. kefyr* (30)	Amphotericin B	28 (93.3)	—	2 (6.7)
	Fluconazole	16 (53.3)	3 (10)	11 (36.7)
	Itraconazole	19 (63.3)	2 (6.7)	12 (40)
	Ketoconazole	16 (53.3)	3 (10)	11 (36.7)

*C. parapsilosis* (18)	Amphotericin B	15 (83.4)	2 (11.1)	1 (5.5)
	Fluconazole	11 (61.1)	2 (11.1)	5 (27.8)
	Itraconazole	10 (55.5)	2 (11.1)	6 (33.4)
	Ketoconazole	11 (61.1)	2 (11.1)	5 (27.8)

*C. guilliermondii* (11)	Amphotericin B	11 (100)	—	—
	Fluconazole	7 (63.6)	2 (18.2)	2 (18.2)
	Itraconazole	6 (54.5)	2 (18.2)	3 (27.3)
	Ketoconazole	8 (72.7)	—	3 (27.3)

*C. dubliniensis* (9)	Amphotericin B	9 (93.3)	—	2 (6.7)
	Fluconazole	3 (33.3)	3 (33.3)	3 (33.3)
	Itraconazole	4 (44.5)	2 (22.2)	3 (33.3)
	Ketoconazole	3 (33.3)	—	6 (66.7)
